# Microglia in Alzheimer’s Disease in the Context of Tau Pathology

**DOI:** 10.3390/biom10101439

**Published:** 2020-10-14

**Authors:** Juan Ramón Perea, Marta Bolós, Jesús Avila

**Affiliations:** 1 Department of Molecular Neuropathology, Centro de Biología Molecular “Severo Ochoa” (CSIC-UAM), 1 Nicolás Cabrera, 28049 Madrid, Spain; jrperea@cbm.csic.es (J.R.P.); mbolos@cbm.csic.es (M.B.); 2 Network Center for Biomedical Research on Neurodegenerative Diseases (CIBERNED), 5 Valderrebollo, 28031 Madrid, Spain

**Keywords:** Alzheimer’s disease, tauopathies, tau, Aβ, microglia, neuroinflammation, ApoE, TREM2, CD33, CX3CR1

## Abstract

Microglia are the cells that comprise the innate immune system in the brain. First described more than a century ago, these cells were initially assigned a secondary role in the central nervous system (CNS) with respect to the protagonists, neurons. However, the latest advances have revealed the complexity and importance of microglia in neurodegenerative conditions such as Alzheimer’s disease (AD), the most common form of dementia associated with aging. This pathology is characterized by the accumulation of amyloid-β peptide (Aβ), which forms senile plaques in the neocortex, as well as by the aggregation of hyperphosphorylated tau protein, a process that leads to the development of neurofibrillary tangles (NFTs). Over the past few years, efforts have been focused on studying the interaction between Aβ and microglia, together with the ability of the latter to decrease the levels of this peptide. Given that most clinical trials following this strategy have failed, current endeavors focus on deciphering the molecular mechanisms that trigger the tau-induced inflammatory response of microglia. In this review, we summarize the most recent studies on the physiological and pathological functions of tau protein and microglia. In addition, we analyze the impact of microglial AD-risk genes (*APOE*, *TREM2,* and *CD33*) in tau pathology, and we discuss the role of extracellular soluble tau in neuroinflammation.

## 1. Introduction

Alzheimer’s disease (AD) is recognized as the most common form of dementia associated with aging [1]. It is the main cause of dependence in older adults and the fifth cause of death worldwide, and it has a great impact on those who suffer from this condition, as well as on their families. The number of individuals with some form of dementia has doubled from 1990 (20.2 million) to the present (47 million), mainly due to population growth and aging. In 2015, dementia accrued associated costs amounting to €758 billion, equivalent to 1.1% of the global gross domestic product. It is estimated that the number of patients with dementia will reach 75 million in 2030 and the costs of this condition €1.86 trillion, a burden that may cause health care systems to collapse [2,3].

Described by Alois Alzheimer in 1906 [4,5], AD is characterized by the presence of senile plaques (comprised of amyloid-β peptide (Aβ) [6,7,8]) and neurofibrillary tangles (NFTs) (formed by the aggregation of hyperphosphorylated tau [9,10,11]) in the brain, causing marked glial activation and loss of neurons and synapses [12]. Based on a body of evidence, in 1992, John Hardy and Gerald Higgins proposed the amyloid hypothesis, stating that Aβ accumulation in the brain gives rise to AD pathogenesis, followed by the appearance of NFTs, neuronal death, and cognitive decline [13]. The rationale for this hypothesis was based on the identification of genetic modifications that altered Aβ production or the capacity of this peptide to aggregate [14].

Although genetic evidence supports the amyloid hypothesis, Aβ accumulation is believed to be a necessary, but not sufficient, condition for AD progression. In this regard, several studies have established a greater relationship between NFTs and AD [15,16,17,18]. Furthermore, other authors demonstrate that Aβ toxicity requires the presence of tau [19,20]. Contrary to what occurs in tau pathology, an extensive accumulation of Aβ can be observed in patients who do not present cognitive decline [21,22]. Likewise, individuals with tau and Aβ show higher levels of specific biomarkers compared to those with only Aβ [23,24]. All these studies suggest that although the presence of Aβ is important in AD pathogenesis, other factors such as tau accumulation or neuroinflammation may be the main causes of neurodegeneration.

## 2. Tau Protein

### 2.1. Expression, Isoforms, and Localization

Tau protein regulates the assembly and stabilization of microtubules [25]. It is expressed mainly in neurons of the central nervous system (CNS), although it is also detected to a lesser extent in oligodendrocytes [26,27,28]. In humans, tau protein is encoded by *MAPT* on chromosome 17q21 [29]. The adult human brain has six isoforms of this protein generated by alternative splicing, and these differ in the number of N-terminal domains and repeated binding domains [30]. Specific mutations in *MAPT* [31], the inhibition of the mitochondrial complex I [32], and the presence of microRNAs [33] and RNA binding proteins [34] are some of the factors that determine splicing.

The subcellular localization of tau varies throughout development. In immature neurons, this protein is distributed in the soma and neurites. Later, when axons are generated and neurons acquire polarity, tau is found mostly in the axonal region [35]. Furthermore, it is also found in dendritic spines [36] and the nucleus [37,38], and it is associated with the plasma membrane through binding to phospholipids [39,40]. Likewise, this distribution is determined by the number of tau N-terminal domains. The 0N and 2N isoforms are found primarily in the soma and axon, while the 1N isoform is generally located in the nucleus [41].

### 2.2. Structure and Post-Translational Modifications of Tau

The structure of tau is divided into the projection domain, which holds the N-terminal domains and is thought to regulate the space and crosslinking between microtubules [42,43], and the microtubule binding domain [44]. There is a proline-rich region between the two domains with which signaling proteins such as Fyn can interact [45].

Tau protein is subject to numerous post-translational modifications: phosphorylation, glycosylation, glycation, isomerization, nitration, ubiquitination, SUMOylation, acetylation, and methylation [46]. Of these modifications, tau phosphorylation is the most recurrent. This process is regulated throughout life, being more predominant during embryonic development than in adulthood [47,48]. However, in some pathologies such as AD, tau is hyperphosphorylated [49]. This fact plays a very important role in regulating the physiological function of tau, since it reduces the binding affinity to microtubules and promotes its aggregation, thus compromising the integrity of neurons [49,50]. Furthermore, hyperphosphorylated tau tends to accumulate in the somatodendritic compartment, where it causes AMPA receptor loss, thereby promoting synaptic dysfunction [51]. It has also been described that the hyperphosphorylation of tau can alter its degradation [52], as well as its interaction with other proteins [53,54,55].

A large number of kinases have the capacity to phosphorylate tau [56]. Glycogen-synthase kinase 3β (GSK3β), a protein involved in the regulation of glucose metabolism, has been recognized as the main tau kinase [57]. Others such as p38 (introduced in the later sections) also phosphorylate tau but at lower efficiency [58]. Furthermore, tau can be dephosphorylated by phosphatases such as PP1, PP5, PP2B, and PP2A [59]. Thus, the regulation of the activity of some of these proteins has been proposed as a possible therapeutic target to combat AD [60].

### 2.3. Tau Aggregation

Tau aggregation is characteristic of several neurodegenerative diseases called tauopathies, including AD [61]. These diseases can be classified into three groups depending on the isoforms detected in the aggregates: 4R (progressive supranuclear palsy, corticobasal degeneration, and argyrophilic grain disease), 3R (Pick’s disease), and 3R + 4R (AD and frontotemporal dementia with parkinsonism-17) [62]. Numerous factors promote tau aggregation, but the main trigger of this phenomenon is still unclear. The observation that tau aggregates are hyperphosphorylated [49] indicates that this post-translational modification plays an important role in this process [63,64]. In this regard, it has recently been shown that specific tau phosphorylation at Ser208 promotes the aggregation of the protein, whereas phosphorylation at Ser202 and Thr205 leads to its mislocalization to the soma and dendrites [65]. However, phosphorylation at specific sites can suppress aggregate formation [66]. Moreover, tau truncation removes some residues, thereby promoting a greater number of tau–tau than tau–tubulin interactions [67]. Additionally, the concentration of tau may be a key factor in the initiation of this process, since high concentrations of this protein favor the formation of paired helical filaments (PHFs) in vitro [68]. Some mutations that reduce the binding affinity of tau to microtubules have also been shown to accelerate aggregate formation [69]. Furthermore, several anionic molecules (glycosaminoglycans and RNA, among others) act as cofactors [70,71].

The different forms of tau presented below have been classified on the basis of their aggregation state [72]. Tau monomers are the most abundant forms in the brain [73]. They are characterized by high solubility and they normally acquire a random structure [74]. Monomers can interact with each other to form dimers, trimers, oligomers (between six and eight molecules), insoluble oligomers (approximately 40 molecules), and insoluble structures [75,76,77] with different folds [78,79,80,81,82].

### 2.4. Main Functions of Tau

In 1975, Weingarten et al. proposed that tau regulates microtubule assembly and stabilization [25]. This function has recently been further explored by Qiang et al., who suggest that tau is not strictly a microtubule-stabilizing protein, but that it rather allows microtubules to have long labile domains [83]. Additionally, tau plays a key role in axonal transport, since it competes with kinesin and dynein motor proteins in their binding to microtubules [84]. Tau is also very important in axonal maturation and elongation, since its silencing inhibits neurite formation [85]. Regarding the regulation of synaptic plasticity, some studies suggest that tau is involved in long-term depression (LTD) and potentiation (LTP) [86,87]. This protein is also involved in adult hippocampal neurogenesis against positive stimuli or acute stress [88], as well as in the integrity of DNA and RNA [89,90].

The *Mapt^−/−^* mouse has been widely used to study the aforementioned functions of tau. This model does not present a severe phenotype throughout its development thanks to compensation by other microtubule-stabilizing proteins [91,92]. During aging, these animals show brain atrophy, cognitive deficits, and motor disturbances [93,94]. These findings indicate that, although tau has a deleterious role in neuropathological processes, its physiological functions are especially important at advanced ages.

### 2.5. Mechanisms of Tau Propagation

Several in vitro [95] and in vivo studies [96,97,98,99] have revealed that tau is transmitted from one cell to another following a stereotypical pattern, thus supporting the tau prion propagation hypothesis in AD and other tauopathies [100]. Although these observations provide solid evidence of this phenomenon, the mechanisms of tau release, internalization, and processing are still being studied.

**Figure 1:**
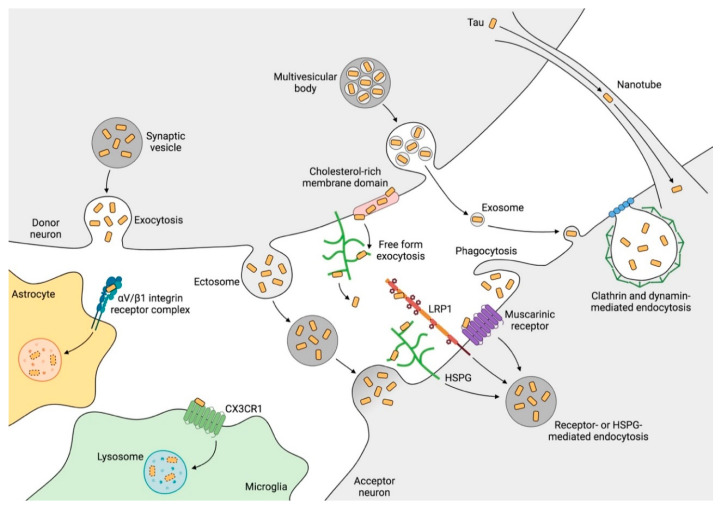
Tau transmission mechanisms. The processes depicted above occur commonly at synapses, where tau can be released to the extracellular space through synaptic vesicles or by direct translocation. Tau is then internalized through receptor- or heparan sulfate proteoglycan (HSPG)-mediated endocytosis, clathrin- and dynamin-mediated endocytosis, and phagocytosis. In addition, tau can be spread through extracellular vesicles (ectosomes and exosomes) that fuse to the membrane of the recipient cell. Moreover, nanotubes establish intercellular communication that serves as a bridge for tau propagation. Microglia and astrocytes also have the ability to internalize and degrade the tau present in the extracellular medium, although the mechanisms involved, especially in astrocytes, remain poorly understood. CX3CR1: fractalkine receptor, HSPG: heparan sulfate proteoglycan, LRP1: low-density lipoprotein receptor-related protein 1. Created with BioRender.com.

Extracellular tau was once thought to originate from axonal degeneration and neuronal death [101,102]. However, neuronal activity regulates tau release physiologically without cell death [103]. It has been suggested that the release mechanism occurs mainly in synapses [104] through synaptic vesicles [103], exosomes [105,106], ectosomes [107], or in a free form [108,109,110]. Once released, tau can be internalized by other neurons [111] through interaction with muscarinic receptors, low-density lipoprotein receptor-related protein 1 (LRP1) or heparan sulfate proteoglycans (HSPGs) [112,113,114,115], clathrin- or dynamin-mediated endocytosis [111,116], macropinocytosis [115], or direct fusion of the vesicles with the membrane. Additionally, tunneling nanotubes allow tau to be transported from one cell to another [117] (Figure 1). Propagation commonly involves the monomer and oligomer forms of tau, which are susceptible to aberrant folding and post-translational modifications such as truncation and phosphorylation [118].

Once internalized, tau interacts with other tau molecules of the receptor neuron and induces their aberrant folding, thus propagating the pathology [116,119]. Propagation does not occur only in neurons as glia are also capable of engulfing extracellular tau [120,121,122,123] and therefore of contributing to tau propagation [124,125,126,127], although these cells have the capacity to degrade it [120,125,128] (Figure 1).

## 3. Microglia

### 3.1. History and Origin of Microglia

More than a century ago, Iliá Méchnikov discovered that the introduction of rose thorns into starfish larvae caused the accumulation of mobile cells around these foreign bodies. This observation led him to conclude that these cells behaved as defense agents. On the basis of this discovery, he expounded the phagocytosis theory, and he tirelessly explored the role of this phenomenon in various diseases [129]. Two decades later, in the brains of rabbits infected with rabies virus, Nicolás Achúcarro visualized cells that had the ability to phagocytize damaged neurons [130]. After the sudden death of Achúcarro, Pío del Río Hortega continued his research and improved the existing staining techniques. These advances allowed him to distinguish that a subpopulation of apolar cells, previously identified by Cajal [131], were actually microglia, making him the first scientist to describe this cell type [132,133,134,135,136,137].

Microglia are the resident macrophages of the CNS and the only cell type, along with endothelial cells, that do not have a neuroectodermal origin. The cells that give rise to microglia leave the yolk sac blood islands at E9.5 to colonize the neuroepithelium, just before the blood–brain barrier (BBB) is formed. Thereafter, this population proliferates and expands throughout the CNS [138,139,140]. However, the coupled proliferation and apoptosis of microglia imply a stable population from postnatal stages until the first signs of aging appear [141]. In fact, it is estimated that 28% of microglia in humans are renewed every year and that the half-life of these cells is 4.2 years [142].

Microglia make up 5–12% of CNS cells, and their distribution in the adult brain varies depending on the region, being more present in the hippocampus, basal ganglia, and substantia nigra compared to in the nervous tracts, cerebellum, and brain stem [143]. Moreover, transcriptomic analysis has allowed the study of the spatial and temporal heterogeneity of microglia, identifying a broad diversity of cellular subtypes depending on age, area, and pathology [144,145,146,147,148,149]. Furthermore, microglia also have sex-specific transcriptomic features. Female microglia show a neuroprotective phenotype, whereas the transcriptome of male microglia is enriched in inflammation-associated genes [150]. Hormonal cues and sex chromosomes are thought to determine these differences [151]. A very recent study has demonstrated that sex-related differences in microRNAs expressed in microglia lead to a differential response of these cells to tau pathology in males and females [152]. This observation, together with other aspects, could partially explain gender differences in the incidence of some neurodegenerative diseases [151].

### 3.2. Physiological Functions of Microglia

Microglia contribute to the early stages of development by establishing the neural architecture of the CNS. To this end, microglia engulf neurons that have been over-generated [153]. This phenomenon is still evident in neurogenic niches in the adult stage [154]. Furthermore, it has recently been discovered that microglia produce a set of neurogenic factors that contribute to the maintenance and correct regulation of adult hippocampal neurogenesis [155].

Furthermore, microglia shape neuronal circuits. In this regard, these cells can eliminate non-functional synapses, thus ensuring the establishment of efficient synaptic contacts [156]. Additionally, this cell type engulfs the extracellular matrix around dendritic spines and contributes to postnatal synapse formation [157,158]. While previous studies have focused on the role of microglia in synapses during the early stages of development, a very recent report demonstrates that microglial processes also maintain direct contact with the soma of neurons, thereby ensuring homeostasis [159]. In addition to their interaction with neurons, microglia support the functions of other cell types such as oligodendrocytes and endothelial cells. In this context, microglia have been shown to play a critical role in axon myelination [160] and in the vascularization of neural tissue [161].

In the adult brain, microglia show a basal motility characterized by continuous extension and retraction of their processes without movement of cell bodies. This motility allows microglia to quickly detect changes in their microenvironment and to rapidly phagocytose harmful substances [162,163]. To do so, these cells are equipped with a set of receptors that allow them to recognize pathogens, misfolded proteins, cytokines, metabolites, inorganic substances, and changes in pH [164]. The factors that regulate microglial motility and morphology are largely unknown, although some purinergic receptors [165], ion channels [166] and neurotransmitters [167] have been implicated.

### 3.3. Contribution of Microglia to Tau Pathology

Alois Alzheimer was the first to recognize the involvement of glia in the disease that would bear his name [168]. However, it was in the 1990s that microglia were shown to interact with Aβ and tau [169,170]. For the last thirty years, the scientific community, supported by the amyloid hypothesis, has focused on investigating the contribution of Aβ to neuroinflammation, while less attention has been paid to the association between microglia and tau pathology.

**Figure 2:**
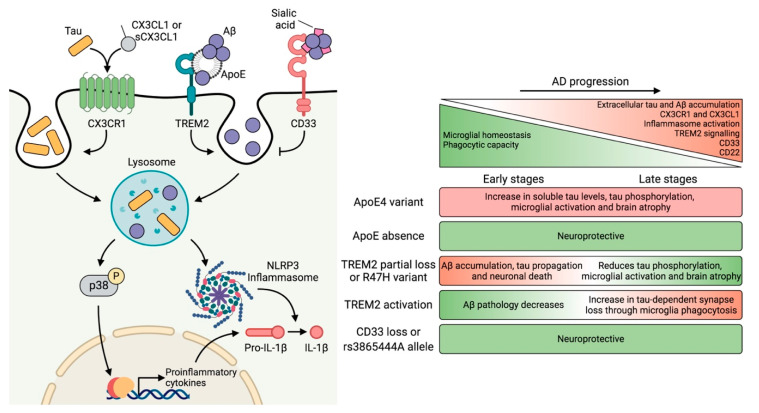
Overview of microglial receptors involved in neuroinflammation. Amyloid-β peptide (Aβ) and tau accumulate in the extracellular space during Alzheimer’s disease (AD). At the same time, CX3CR1 (fractalkine receptor) and CX3CL1 (fractalkine) levels increase, which compromises this communication axis, thus altering microglial homeostasis. Moreover, the increasing levels of CD33 and CD22 reduce the phagocytic capacity of these cells. In the extracellular space, tau competes with CX3CL1 for its binding to CX3CR1, a receptor involved in tau internalization. Moreover, Aβ directly interacts with triggering receptor expressed on myeloid cells 2 (TREM2) or through apolipoprotein E (ApoE), promoting its phagocytosis. Conversely, Aβ can also bind to CD33 via sialic acid residues, thereby preventing phagocytosis. It has been described that tau binds to ApoE and that tau aggregates contain sialic acid residues. However, the interaction of tau with these receptors has not yet been described. Both Aβ and tau promote the activation of the p38 Mitogen-activated protein kinase (MAPK) pathway and the NOD-, LRR-, and pyrin domain-containing 3 (NLRP3) inflammasome, which trigger the production of proinflammatory cytokines. The table on the right summarizes the effects of genetic variants highly expressed in microglia that confer susceptibility to AD. The absence of ApoE and CD33 confers neuroprotection. In contrast, the effects of TREM2 depend on the stage of the disease. For this reason, promoting TREM2 activation is effective in the preclinical phase against Aβ pathology. However, at advanced stages, TREM2 signaling is detrimental as it facilitates the progression of the disease in the context of tau pathology. AD: Alzheimer’s disease, ApoE: apolipoprotein E, Aβ: amyloid-β, CD: cluster of differentiation, CX3CL1: fractalkine, CX3CR1: fractalkine receptor, IL-1β: interleukin-1β, NLRP3: NOD-, LRR-, and pyrin domain-containing 3, Pro-IL-1β: interleukin-1β (inactive precursor), sCX3CL1: soluble fractalkine, TREM2: triggering receptor expressed on myeloid cells 2. Created with BioRender.com.

Virginia Lee’s group developed one of the most extensively used mouse models of tauopathy (P301S). They observed that treatment of this animal with an immunosuppressant inhibited microglial activation and attenuated tau pathology [171]. Those authors postulated that microgliosis precedes tau pathology. However, other studies report that tau propagation is a very early phenomenon that triggers microglial activation [172,173]. Later, it was shown that activated microglia played an important role in the propagation of tau [124]. Subsequently, it was demonstrated that tau was phagocytosed by microglia [120,121]. However, the molecular mechanism through which microglia promoted tau pathology was unknown. It has recently been described that tau induces NOD-, LRR-, and pyrin domain-containing 3 (NLRP3) inflammasome activation [174], a multimeric signaling complex that leads to the activation of inflammatory processes through IL-1β proteolysis. Similarly, NLRP3 activation is also mediated by Aβ (Figure 2). Therefore, inflammasome deletion reduces the formation of NFTs and senile plaques [174,175,176].

Aging is the most important risk factor in AD [177]. In this context, microglia are believed to promote the progression of the pathology through a decrease in their neuroprotective functions, an increase in their toxicity, and alterations in their response to several stimuli, thus giving rise to a senescent phenotype [178]. These age-associated changes have been previously characterized in microglia and include alterations in cytokine secretion [179], an increase in the expression of activation markers [180], and the appearance of dystrophic morphologies [181]. It has recently been proposed that the elimination of senescent microglia and the repopulation of the CNS with new microglia, capable of supplying the functions of the former, may provide a beneficial therapeutic strategy for AD [182]. In this regard, pharmacological depletion of microglia has been shown to block tau propagation [183] and neurodegeneration [184]. Furthermore, another study demonstrated the presence of senescent markers in glia from the P301S mouse. The depletion of senescent cells in this model prevented gliosis, tau hyperphosphorylation, and neuronal degeneration, thereby preserving cognitive function [185]. Similarly, this strategy reduced senile plaque formation in an Aβ mouse model [186,187].

## 4. Genetic Risk Factors and Their Impact on Microglial Function

### 4.1. APOE

The hypothesis that activation of the innate immune system contributes to the pathogenesis of AD has been supported by genome-wide association studies, which have identified a set of genetic variants that are highly expressed in microglia. Among these, the *ε4* variant of *APOE* is the strongest genetic risk factor in AD [188,189] and it is associated with an enhanced innate immune response [190]. In the brain, apolipoprotein E (ApoE), which is secreted primarily by microglia and astrocytes, acts as a transporter of lipoproteins between cells [27,28,191]. Several groups have reported that ApoE is also involved in Aβ metabolism [192,193,194], and subsequent studies have shown that it mediates the microglial response to amyloid pathology [195].

Although the interaction between ApoE and tau was described almost three decades ago [196], its contribution to tau pathology has been addressed only recently. In this regard, the *ε4* variant in the P301S mouse increases soluble tau levels, tau phosphorylation, microglial activation, and brain atrophy. However, the absence of ApoE is neuroprotective since it prevents neurodegeneration and inflammation [197]. Additionally, a more recent study led by the same group indicates that ApoE regulates neurodegeneration in the P301S mouse by regulating the microglia-mediated innate immune response [198] (Figure 2).

Furthermore, ApoE4 binds inefficiently to LRP1 in pericytes, and the absence of signaling triggers the activation of the cyclophilin-A/NF-κB/MMP9 cascade, causing the breakdown of the BBB [199,200]. This disruption leads to the extravasation of proteins such as fibrin, which induces microglia-mediated spine elimination and cognitive decline [201].

### 4.2. TREM2

The triggering receptor expressed on myeloid cells 2 (TREM2) is another molecule that interacts with ApoE [202,203]. Krasemann et al. described that this interaction shifts microglia to a neurodegenerative phenotype and suggested that targeting the TREM2–ApoE pathway may restore homeostatic microglia in amyloid pathology [204].

The R47H variant confers TREM2 loss-of-function and it is the second main genetic risk factor in AD in terms of the magnitude of its effects [205]. Expressed in microglia, TREM2 is a transmembrane receptor that induces phagocytosis, modulates inflammatory signaling, and promotes survival [206]. Partial or complete TREM2 absence increases Aβ accumulation and neuronal death as this receptor modulates microglial response against these aggregates [207]. Moreover, TREM2 deficiency impedes microglia clustering around Aβ deposits, allowing senile plaques to become more diffuse and therefore increasing neuronal damage [208]. Conversely, TREM2 activation with an agonistic antibody reduces amyloid pathology [209].

The contribution of TREM2 to tau pathology offers opposing results. One of the first reports showed that the absence of TREM2 increases tau hyperphosphorylation and aggregation (due to c-Jun N-terminal kinase (JNK), Extracellular signal-regulated kinase (ERK), p38, and GSK3β activation) and also induces microglial activation in the hTau mouse [210]. Subsequently, another study postulated that lack of TREM2 attenuates neuroinflammation and prevents neuronal death in the P301S mouse [211]. Later, the same group observed that TREM2 deficiency or the R47H variant induces tau seeding and propagation in APP/PS1 mice subjected to the stereotaxic injection of tau from AD patients. They reported that microglial impairment in these animals promotes NFT accumulation around senile plaques, a phenomenon that also occurs in patients with *TREM2* mutations [212]. Nonetheless, a novel study revealed that the R47H variant has a neuroprotective role by reducing brain atrophy, synapse loss, tau phosphorylation, microglial activation, and the engulfment of postsynaptic elements in the P301S mouse. The authors of that study explained that the R47H variant attenuates microglial phagocytosis, thereby leading to a lower synapse loss and, consequently, less brain atrophy. If the R47H variant increases the risk of AD, the question arises as to why it is protective against tau pathology. They pointed out that the role of TREM2 also depends on Aβ pathology and the stage of the disease. At early phases, the R47H variant reduces microgliosis around senile plaques, thereby increasing their number, and also promotes tau propagation. However, in advanced stages of the disease, when tau pathology is conspicuous, the variant attenuates tau-dependent synapse loss by reducing microglial phagocytosis [213].

Some of the discrepancies found in these studies could be explained by the mouse model used, the differences between murine and human TREM2, and the stage of the disease. Regarding the latter, TREM2 appears to have a dual role throughout pathology, and its targeting might not be straightforward. In this regard, TREM2 function should be potentiated at early time points. However, its activity should be partially suppressed in advanced stages since complete abolishment could lead to Nasu–Hakola disease, which is characterized mainly by bone lesions [214] (Figure 2).

Additionally, soluble TREM2 (sTREM2) is one the of most reliable immune biomarkers in AD [215]. Most sTREM2 is produced by ADAM10 proteolysis, and this phenomenon has been linked to an increase in TREM2 signaling, thus suggesting that this receptor exerts a protective role [216,217]. A further report also led by Christian Haass showed that increased levels of sTREM2 are associated with tau pathology [24]. More research is still needed to establish whether sTREM2 levels correspond to the activity of this receptor. If confirmed, TREM2 cleavage emerges as an approach to reduce TREM2 levels in the membrane of microglia to halt the activation of these cells during the disease.

### 4.3. CD33

*CD33* is another AD risk gene [218,219,220] that encodes for a transmembrane receptor expressed in microglia [27,28]. As a member of the sialic acid–binding immunoglobulin-like lectins (Siglecs) [221], CD33 recognizes sialic acid residues of proteins and lipids triggering a signaling cascade, which suppresses the immune response [222].

*CD33* expression is increased in AD, thereby causing a reduction in microglial activation and Aβ clearance. This reduction occurs due to the interaction of sialic acid residues of Aβ with CD33, leading to the inhibition of Aβ internalization. However, knockout of *CD33* in the APP/PS1 mouse promoted Aβ uptake by microglia, thus reducing the amyloid plaque burden [223]. This reversal was also achieved in 5×FAD mice, which also showed an improvement in cognitive function. Conversely, TREM2 deletion in these animals abrogated this protective effect. Transcriptomic data revealed that gene expression changes caused by CD33 deletion were dependent on TREM2 presence. In contrast, gene expression changes caused by TREM2 deletion occurred regardless of CD33. Therefore, the authors of that work proposed that TREM2 acts downstream of CD33 [224]. Thus, CD33 inhibition and TREM2 activation emerge as a promising strategy, at least during the early stages of AD (Figure 2).

Furthermore, NFTs, along with other histopathological hallmarks that contain hyperphosphorylated tau, also present sialic acid residues [225,226]. In this regard, it would be interesting to explore whether tau interacts with CD33, as well as to analyze the role of this receptor in tauopathies.

## 5. Neuron–Microglia Crosstalk: Implications of the CX3CL1–CX3CR1 Axis and Downstream Signaling

Of the cell–cell communicate strategies, the use of chemokines is one of the most highly regulated systems. Normally, a single cell type expresses the ligand for a receptor that is selectively found in another cell population, thereby conferring a high degree of specificity to the resulting signaling axis [227]. This is the case of fractalkine (CX3CL1, C-X3-C chemokine ligand 1), a chemokine produced by neurons that binds to its receptor (CX3CR1, C-X3-C chemokine receptor 1), which is specifically expressed in microglia [27,28,228,229]. CX3CL1 can be either membrane-bound or soluble (sCX3CL1) after cleavage of its N-terminal domain. The membrane-bound form acts as an adhesion molecule, while sCX3CL1 responds as a chemoattractant, promoting cell migration [230]. CX3CR1 belongs to the G protein-coupled receptor family. The inhibitory G subunit prevents cyclic adenosine monophosphate (cAMP) production, thereby giving rise to a series of second messengers known to regulate a broad repertoire of cellular functions such as transcription, migration, proliferation, and apoptosis [231,232,233].

In addition to being essential in CNS development and connectivity [233], the CX3CL1–CX3CR1 axis is in the first line of defense in response to neuronal damage and neuroinflammation, exerting neuroprotective signaling [234,235]. During AD, CX3CR1 and CX3CL1 levels increase in the brain, thereby compromising microglial homeostasis and neuronal circuits [236,237,238]. The generation of the *Cx3cr1^−/−^* mouse, together with its crossing with other AD models, has greatly contributed to our understanding of the role of the CX3CL1–CX3CR1 axis in this pathology. Moreover, CX3CR1 deficiency decreases Aβ accumulation due to an increase in the phagocytic capacity of microglia [239]. Conversely, the absence of this receptor in a mouse model of tauopathy increases tau hyperphosphorylation and aggregation and amplifies microglial activation, thereby causing IL-1β secretion [124,240]. By activating p38 in neurons, this cytokine promotes tau hyperphosphorylation, thereby worsening the pathology [241]. Subsequently, tau phagocytosis was shown to be reduced in microglia lacking CX3CR1. This observation may be due to the fact that the interaction between tau (which possesses a 37% amino acid sequence identity with CX3CL1) and CX3CR1 promotes its internalization [237] (Figure 2). In this regard, two recent studies support these results. The first one determined that *Cx3cr1^−/−^* microglia present a lower phagocytic and migratory capacity per se [242], while the second revealed that *Cx3cr1^−/−^* microglia of young animals are very similar to the wild type (WT) microglia of older animals [243]. On the basis of their findings, the authors of the latter study suggested that the absence of CX3CR1 confers a premature aging transcriptome to these cells, accompanied by a decrease in phagocytosis. These findings pose the question as to why the absence of CX3CR1 promotes the phagocytosis of Aβ and not of tau. One possibility is that the absence of CX3CR1 signaling enhances the synthesis of Aβ receptors and enzymes that degrade this peptide [244]. In turn, this pathway may block those corresponding to tau, thus having opposite effects on each pathology.

In addition, some groups have focused on analyzing the role of CX3CL1 in mouse models of tauopathy. In this regard, sCX3CL1 overexpression reduces neuroinflammation and tau pathology in the rTg4510 mouse [245]. In contrast, Bemiller et al. showed the opposite results in an hTau mouse and concluded that sCX3CL1 is not protective. The authors of that work suggested that the discrepancies between these studies are due to the experimental approach (recombinant adeno-associated virus (rAAV) vector-mediated overexpression vs. transgenic mouse), the differences in sCX3CL1 structure (presence or not of mucin stalk), and the tauopathy mouse model used (rTg4510 vs. hTau) [246]. However, the CX3CL1 intracellular fragment enhances neurogenesis, improves cognitive function, and increases the lifespan. Nevertheless, its effects on tau pathology are minimal [247,248].

In brief, all the studies mentioned above highlight the importance of the CX3CL1–CX3CR1 signaling pathway in neuron–microglia crosstalk, as well as in the progression of AD.

## 6. Extracellular Soluble Tau as the Main Driving Force of Toxicity

Numerous authors state that tau aggregates are not as toxic as previously believed and that their formation may suppose a neuroprotective event [72]. Added to this, it has been shown that neuronal death does not depend on the presence of NFTs [249]. Likewise, neurons bearing NFTs can survive up to 20 years [250]. Consequently, these aggregates have been found in healthy individuals and the neurons that carry them are correctly integrated into the cortical circuit [251]. Furthermore, synaptic, cognitive and behavioral alterations occur before NFTs are formed [252,253,254,255]. More recent studies have revealed that soluble tau blocks Aβ-dependent hyperactivity, thereby leading to impaired neuronal circuit function despite the presence of NFTs [256,257]. Additionally, several soluble tau species (which differ in seeding activity, phosphorylation, and oligomerization) rather than tau accumulation have been described to determine the heterogeneity in AD progression [258]. All these reports suggest that soluble tau species are the main protagonists of AD, which would explain why therapeutic strategies against Aβ or aggregated tau have failed. However, clinical trials focused solely on tau may need to be reconsidered as some authors propose the need for a deeper study of the interrelationship between Aβ and tau [259].

Other studies also support the importance of soluble dephosphorylated tau in neurodegenerative diseases. Thus, there is some evidence supporting the toxicity of dephosphorylated tau resulting from the action of extracellular phosphatases such as tissue-nonspecific alkaline phosphatase (TNAP). In this regard, previous reports showed that TNAP, whose expression and activity increases in AD, is capable of removing phosphorylated residues of tau present in the extracellular space. Afterward, tau acts as an agonist for muscarinic receptors, increasing the amount of Ca^2+^ and, thus, causing neuronal death. Furthermore, TNAP expression and activity increases after tau addition to a neuron culture medium, thereby creating a positive feedback loop [102,112,260,261]. Additionally, exome sequencing analysis has recently identified genetic variants of some phosphatases that confer protection against AD [262]. However, the role of these variants in tau dephosphorylation remains unexplored.

As we previously mentioned, tau also interacts with glia [120,121,122,123,126,169]. In this regard, a wide repertoire of receptors present in microglia that interact with Aβ are known [263]. In contrast, the analysis of the tau interactome has not yet identified the binding of tau to any of these receptors [264,265,266]. Nonetheless, our group described that tau interacts with CX3CR1 in microglia [237]. In addition, a more recent study has shown that tau binds to an integrin receptor in astrocytes [267] (Figure 1). In this respect, biotinylation-based proximity methods such as the one applied in that study could reveal more candidates. In fact, there is previous evidence about the interaction of tau with the plasma membrane [39]. Furthermore, the association of tau with this cell structure is regulated by its phosphorylation state [268], denoting that the dephosphorylation of tau increases its interaction with the membrane [269] and vice versa [270]. Consequently, it is plausible that membrane-bound tau interacts with receptors of the innate immune system such as Toll-like receptors (TLRs) [271], thereby reinforcing the hypothesis of the toxicity of dephosphorylated extracellular tau. In this context, we found that dephosphorylated tau activates p38 MAPK in microglia, triggering a proinflammatory response [272] (Figure 2). Interestingly, previous reports in which non-phosphorylated tau underwent a phosphorylation reaction demonstrated that phosphorylated tau is less internalized by microglia [237]. Accordingly, it could be considered that phosphorylated residues mask the binding region of tau, or alter its conformation, preventing its interaction with receptors involved in tau phagocytosis and/or p38 activation.

Therefore, these studies support the hypothesis that extracellular dephosphorylated tau has a toxic effect on surrounding cells such as microglia, whereas aggregated and/or hyperphosphorylated intracellular tau plays a key role in neuron functionality.

## 7. Future Perspectives

The aforementioned studies highlight that innate immune activation is a key factor in aging and AD progression. In this context, more research is needed to identify the role of other genetic risk factors, as well as the epigenetic changes that principally occur in microglia [273,274,275]. Moreover, the contribution of the adaptive immune system to aging and AD progression remains largely unexplored. In this regard, it has been found that a subpopulation of CD8^+^ T cells patrols the CNS [276]. These cells have also been detected in APP/PS1 mice but barely in hTau animals [277]. Therefore, it would be intriguing to investigate which specific antigen this cell population recognizes.

In addition, the study of gut microbiota in neurodegenerative diseases has attracted the attention of the scientific community. Several reports indicate that microbiota govern the maturation and function of microglia [278] and, thus, regulate Aβ phagocytosis [279,280,281]. However, the role of this assemblage of microorganisms in tau pathology is unknown.

Regarding microglial malfunction during aging, it has been shown that microglial repopulation reverses synaptic alterations and improves cognitive function in aged mice [282]. Furthermore, CD22 (whose levels increase with age) has emerged as a suppressor of microglial phagocytosis in these animals (Figure 2). Nevertheless, blockade of CD22 restores phagocytosis, and animals showed cognitive improvement [283]. Given these observations, it would be of interest to determine whether these two strategies can improve the ability of microglia to internalize tau protein.
